# Supplementary material for: PAI-1 Inhibitor TM5441 Attenuates Emphysema and Airway Inflammation in a Murine Model of Chronic Obstructive Pulmonary Disease
Source: Int J Mol Sci. 2025 Jul 23;26(15):7086. doi: 10.3390/ijms26157086 (PMC12345759; doi:10.3390/ijms26157086)
Supplement: Supplementary file 1 [file ijms-26-07086-s001.zip › ijms-3752502-Supplementary 07212025.pdf]

Additional files

Additional file 1: Figure S1. Flow cytometry histograms and plots illustrating the gating strategy employed.

A) Representative histogram overlays are shown, depicting fully stained samples (black) and fluorescence-minus one (FMO) controls (gray). FMO controls were used to establish gating thresholds.

B) Representative flow cytometry plots are shown. Events were gated sequentially as follows: debris was excluded based on forward and side scatter (FSC-A and SSC-A). As no viability dye was used, dead cells were not specifically excluded. The main cell population was defined based on size and granularity. Singlets were selected by comparing FSC-H to FSC-A, and CD45+ leukocytes were subsequently gated. Immune cell subsets were defined as follows: macrophages were identified as $CD45^+$ Siglec-F^+ $CD11c^+$ cells, and neutrophils as $CD45^+$, Ly-6G/Ly-6C $(Gr\text{-}1)^+$, $CD11b^+$ cells.
FSC, forward scatter; SSC, side scatter; Gr-1, Ly-6G/Ly-6C
