# Supplementary figures and images for: PAI-1 Inhibitor TM5441 Attenuates Emphysema and Airway Inflammation in a Murine Model of Chronic Obstructive Pulmonary Disease
Source: Int J Mol Sci. 2025 Jul 23;26(15):7086. doi: 10.3390/ijms26157086 (PMC12345759; doi:10.3390/ijms26157086)

Figure S1. Flow cytometry histograms and plots illustrating the gating strategy employed

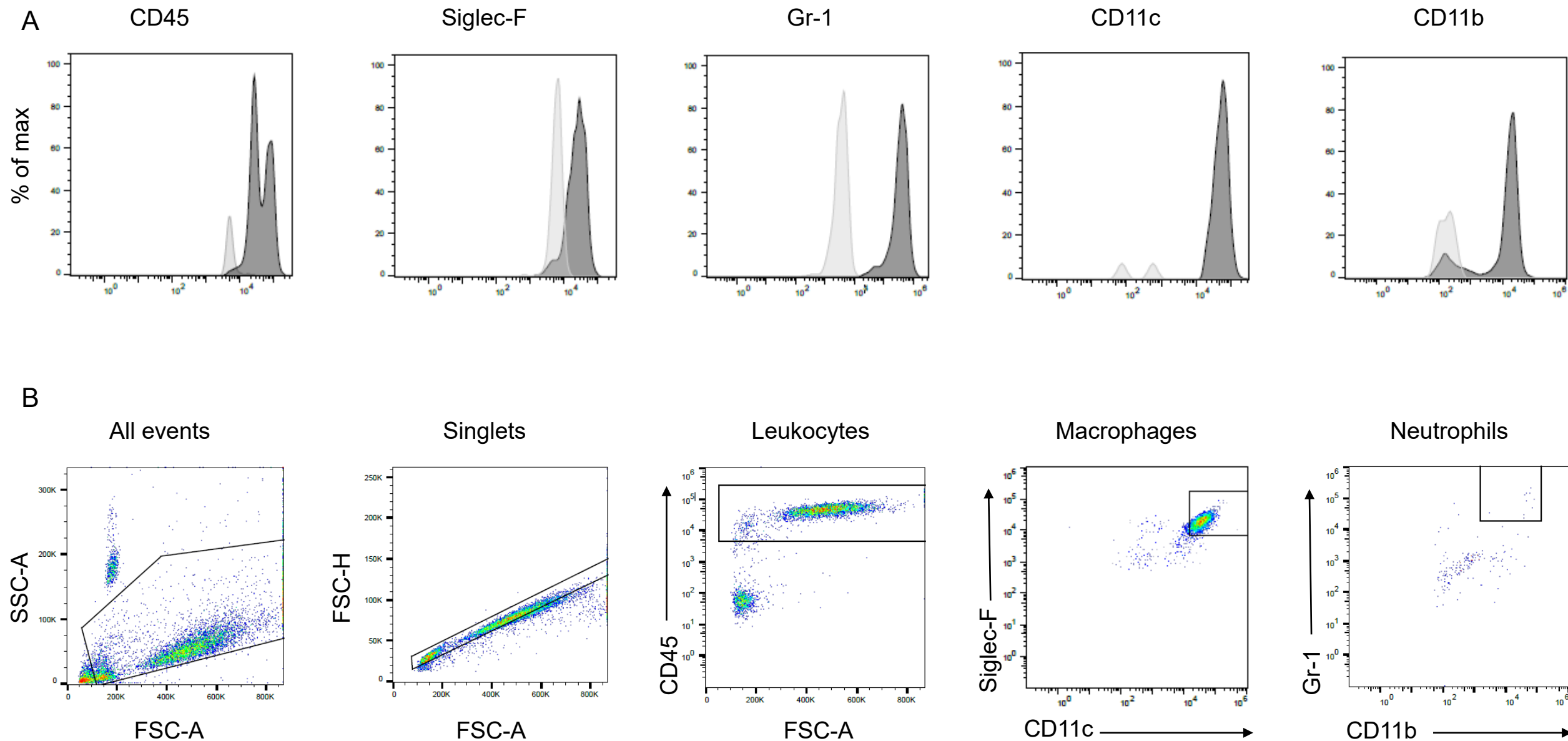

Supplement: Supplementary file 1 [file ijms-26-07086-s001.zip › ijms-3752502-Figure S1 07212025 oishi.pdf]
